# Chitosan/platinum nanocubes/Mn(TPDCA)_2_-modified glassy carbon electrodes for the electrochemical quantification of amlodipine in unprocessed plasma samples

**DOI:** 10.1186/s13065-024-01361-6

**Published:** 2024-12-18

**Authors:** Saeedeh Khadivi-Derakhshan, Mohammad Abbasi, Amirhossein Akbarzadeh, Mahtab Pirouzmand, Jafar Soleymani

**Affiliations:** 1 Department of Inorganic Chemistry, Faculty of Chemistry, University of Tabriz, Tabriz, Iran; 2 Pharmaceutical Analysis Research Center, Tabriz University of Medical Sciences, Tabriz, Iran; 3 Liver and Gastrointestinal Diseases Research Center, Tabriz University of Medical Sciences, Tabriz, Iran; 4 Cardiovascular Research Center, Tabriz University of Medical Sciences, Tabriz, Iran

**Keywords:** Chitosan, Platinum nanocubes, Electrochemical detection, Amlodipine, Plasma sample

## Abstract

A novel electrochemical probe is developed to detect amlodipine (AMD) in unprocessed plasma samples. The fabrication process involves the synthesis of platinum nanocubes (Pt NCs) and Mn(TPDCA)_2_ complexes, which are then immobilized them onto the glassy carbon electrode (GCE) surface. The developed electrochemical probe demonstrates exceptional detection performance, with a wide dynamic range, outstanding selectivity, and commendable reproducibility. The linear range and lower limit of detection of the developed method are 53 nM-3.5 µM and 53 nM, respectively. Electrochemical experiments have been conducted to study the kinetics of electrooxidation on the modified electrode, revealing that the process is diffusion-controlled. Furthermore, method validation studies are performed to assess the accuracy, precision, and selectivity of the sensor, demonstrating excellent performance in all these aspects. Consequently, it can be concluded that the sensor is highly suitable for practical applications in drug analysis.

## Introduction

The natural polysaccharide chitosan (CS) has drawn a lot of interest because of its exceptional film-forming ability, biocompatibility, and biodegradability [1]. The chitosan molecules can interact with various materials to produce more stable composites [2]. A variety of nanometer-sized materials, including AuNPs, AgNPs, PtNPs, carbon-based materials, etc., have been used along with CS to build various types of sensory systems [3]. In the fabrication of electrochemical probes, CS is utilized for the main purpose i.e. creating a scaffold for physical adsorption, and to provide a layer with functional groups for the next surface modifications. Metallic nanoparticles, such as Pt NPs, are incorporated into CS-modified surfaces to enhance electrochemically active surface areas and significantly expedite molecule-electrode electron transfer through a signal amplification mechanism [4]. Pt NCs are used in sensing platforms due to their significant surface area and catalytic activity [5].

Hypertension is one of the major risks associated with stroke, coronary heart disease, renal failure, and diabetes [6]. The number of hypertensive patients is growing as a result of living standards [6, 7]. Several categories of pharmaceutical drugs, such as thiazide diuretics, angiotensin II receptor antagonists, calcium channel blockers, and beta-blockers are employed for the treatment of hypertension. One of the medications used to treat hypertension is 3-ethyl 5-methyl 2-[(2-aminoethoxy)methyl]-4-(2-chlorophenyl)-6-methyl-1,4-dihydropyridine-3,5-dicarboxylate (Amlodipine; AMD), which is a dihydropyridine derivative and acts as a third-generation calcium channel blockers [7]. The therapeutic drug monitoring (TDM) concentration of AMD is 5.0 µg/L which is determined after administrating 10 mg of drug in the healthy persons [8]. AMD improvs blood circulation by dilating blood arteries [8]. It slows the gradual narrowing of the arteries by selectively reducing the development of arterial vascular smooth muscle cells and minimizing coronary spasms. This process enhances the blood flow, and provids more oxygen to myocardial tissue [9]. An excessive level of AMD leads some negative side effects such as nausea, fatigue, swelling, and abdominal pain [4]. Hence, the concentrations of AMD must be monitored.

Several techniques, including high-performance liquid chromatography (HPLC) with different detectors [10–12], mass spectroscopy [13], gas chromatography (GC) [14], capillary zone electrophoresis (CE) [15], spectrofluorometric procedures [16], spectrophotometric, Raman spectroscopy [17], and electrochemical method [18–20] were utilized to detect AMD in biological and pharmaceutical media. Recent advancement in the developpments of electrochemical methods have improved the affordability, speed of detection, simplicity, sensitivity, and selectivity. The fabrication of modified electrodes with nanomaterials has received significant attention in designing of sensing probes for different analytes [3]. New advanced materials offer some outstanding features by enhancing the specific surface area, proposing as primary substrates in different disciplines [21–26]. Additionally, the properties of these materials could be improved by applying various surface functionalization materials and methods [27–29].

Different materials have been used for the electrochemical detection of AMD including reduced graphene oxide (rGO-CuS) [30], molecularly imprinted poly(aniline-*co*-anthranilic acid) [3], Pd-Ag/rGO [31], porous NiMoO_4_/CS [32], M-CeO_2_ [33], Ag-Ce_2_(WO_4_)_3_ [7], carboxylated carbon nanotubes (COOH-CNTs)/Ag/NH_2_-CNTs [34], grass-like Pt-doped NiCo_2_O_4_ [4], AlV_3_O_9_/CNTs, tetracarboxylic manganese phthalocyanine-ZnO [35], and TiO_2_NPs-AuNPs-CS [36].

In this study, a glassy carbon electrode (GCE) was modified with Mn(TPDCA)_2_/CS@Pt NCs to fabricate a novel probe for identifying AMD in plasma samples. Initially, Pt NCs and Mn(TPDCA)_2_ complexes were synthesized. Next, CS and Pt NCs were electropolymerized onto the working electrode surface. Finally, the Mn(TPDCA)_2_ complex was immobilized on the surface of the CS@Pt NCs/GCE using a dropping cast approach. The resulting sensor displayed outstanding electrochemical performance in the detection of AMD. It displayed a wide range of detection, high specificity, and reproducibility, making it a highly suitable probe for drug analysis applications.

## Experimental section

### Substances

L-cysteine (C_3_H_7_NO_2_S, 99%), ethylene glycol (EG, C_2_H_6_O_2_, 99.2%), citric acid monohydrate (C_6_H_8_O_7_.H_2_O, 99.5%), and MnCl_2_.2H_2_O (98%) were obtained from Merck Co. (Germany). AMD was purchased from Behestan Darou pharmaceutical company (Tehran, Iran). CS (C_56_H_103_N_9_O_39_, 98.1%), sodium hexachloroplatinate (IV) hexahydrate (Na_2_PtCl_6_.6H_2_O, 98.0%), polyvinylpyrrolidone (PVP, MW 55,000), sodium dihydrogen phosphate (NaH_2_PO_4_, 98.5%), disodium hydrogen phosphate (Na_2_HPO_4_, 98.8%), sodium hydroxide, and sulfuric acid were purchased from Sigma/Aldrich (Germany). To prepare phosphate buffer, NaH_2_PO_4_ and Na_2_HPO_4_ salts were employed. The stock solutions of AMD (20 µg/mL), CS (1000 µg/mL), and Mn(TPDCA)_2_ (1000 µg/mL) were prepared in distilled and stored at 4 °C. The frozen blank plasma samples were obtained from the Blood Transfusion Organization of East Azerbaijan province (Tabriz, Iran). Also, the patient’s plasma samples were collected from Shahid Madani Hospital with an ethical committee license of IR.TBZMED.REC.1402.724.

### Apparatus

The electrochemical measurements were performed using a Metrohm Autolab electrochemical system, with NOVA 2.1 management software (Netherlands) for experimental data analysis. Electrochemical measurements were conducted utilizing the Ag/AgCl (in saturated KCl), platinum wire, and GCE as reference, counter, and working electrodes, respectively. The GCE with a diameter of 2 mm was purchased from Azar Electrode Co. (Iran). The measurements were taken within a potential range of -0.6 to 0.2 V, with a modulation amplitude of 0.025 V, a modulation period of 0.05 s, a time interval of 0.5 s, and a scan rate of 100 mV/s. The voltammograms were recorded utilizing differential pulse voltammetry (DPV) and cyclic voltammetry (CV). The surface morphology of the electrode was investigated using field emission scanning electron microscopy (FE-SEM; Tescan, MIRA3 FEG-SEM, Czech) and atomic force microscopy (AFM; Nanosurf, Nanosurf mobile s, Switzerland). The energy dispersive X-ray analysis (EDX) spectra and elemental mapping on the coated electrodes were recorded with a (Tescan, MIRA3 FEG-SEM, Czech Republic). The average size and ζ-potential of the materials were determined by dynamic light scattering (DLS; Microtrac, Nanotrac wave, UK).

### Synthesis

#### Synthesis of Mn(TPDCA)2

Briefly, citric acid and L-cysteine solutions were mixed in equal amounts and evaporated at 140 °C for 12 h and 80 °C for 24 h to synthesize the 5-oxo-2,3-dihydro-5 H- [1, 3]-thiazolo [3,2-a] pyridine-3,7-dicarboxylic acid (TPDCA) ligand by one-pot technique. Finally, the TPDCA ligand and MnCl_2_.2H_2_O were refluxed at 95 °C for 24 h to prepare the Mn(TPDCA)_2_ complex [37].

#### Synthesis of Pt NCs

The PVP-capped Pt NCs were produced using a recently published method with some modifications [38]. Briefly, 20 mg of KBr and 40 mg of PVP were added to about 3.5 mL of EG and heated up to 180 °C in an oil bath under magnetic stirring for 10 min. After that, 0.5 mL of Na_2_PtCl_6_.6H_2_O solution (40 mg/mL, in EG) was added to the as-prepared solution. After 20 min of reaction time, the liquid was quickly chilled with an ice-water bath. Finally, the PVP-capped Pt cubes were centrifuged and washed twice with acetone and deionized (DI) water. The PVP-capped Pt cubes solution was stored in a refrigerator until use.

### Preparation of the modified GCE

#### Electrode pretreatment and cleaning

The initial phase of electrode preparation involves the cleaning of the surface of them. This process involved the physical cleaning of the GCE electrode through polishing on a pad, followed by washing with both acetone, water/acetone, and DI water, respectively. To further advance the cleaning procedure, the electrode was immersed in an acidic solution consisting of H_2_SO_4_ (50 mM) and HNO_3_ (50 mM). This step was maintained for a minimum duration of 10 min, ensuring the completion of the cleaning process. Next, the electrode was rinsed with DI water and allowed to dry at room temperature. To chemically clean the surface of GCE, it was immersed in a solution of H_2_SO_4_ (100 mM) and subjected to CV technique for 20 cycles within a potential range spanning from − 1.5 to 1.5 V to receive the repetitive CVs.

#### Surface modification

GCE was introduced to a cell containing CS (1000 mg/L) and Pt NCs solutions. The CV technique was employed to electrochemically polymerize CS@Pt NCs on the surface of GCE. The CV was performed for 5 cycles within a potential range of -1.5 to 1.5 V with a scanning rate of 50 mV/s. The CS@Pt NCs modified GCE was further modified by dropping 5 µL of Mn(TPDCA)_2_ dispersion and allowing it to dry at room temperature. Finally, Mn(TPDCA)_2_/CS@Pt NCs-modified GCE was used for detecting AMD in plasma samples.

## Results and discussions

### Characterizations

**Fig. 1:**
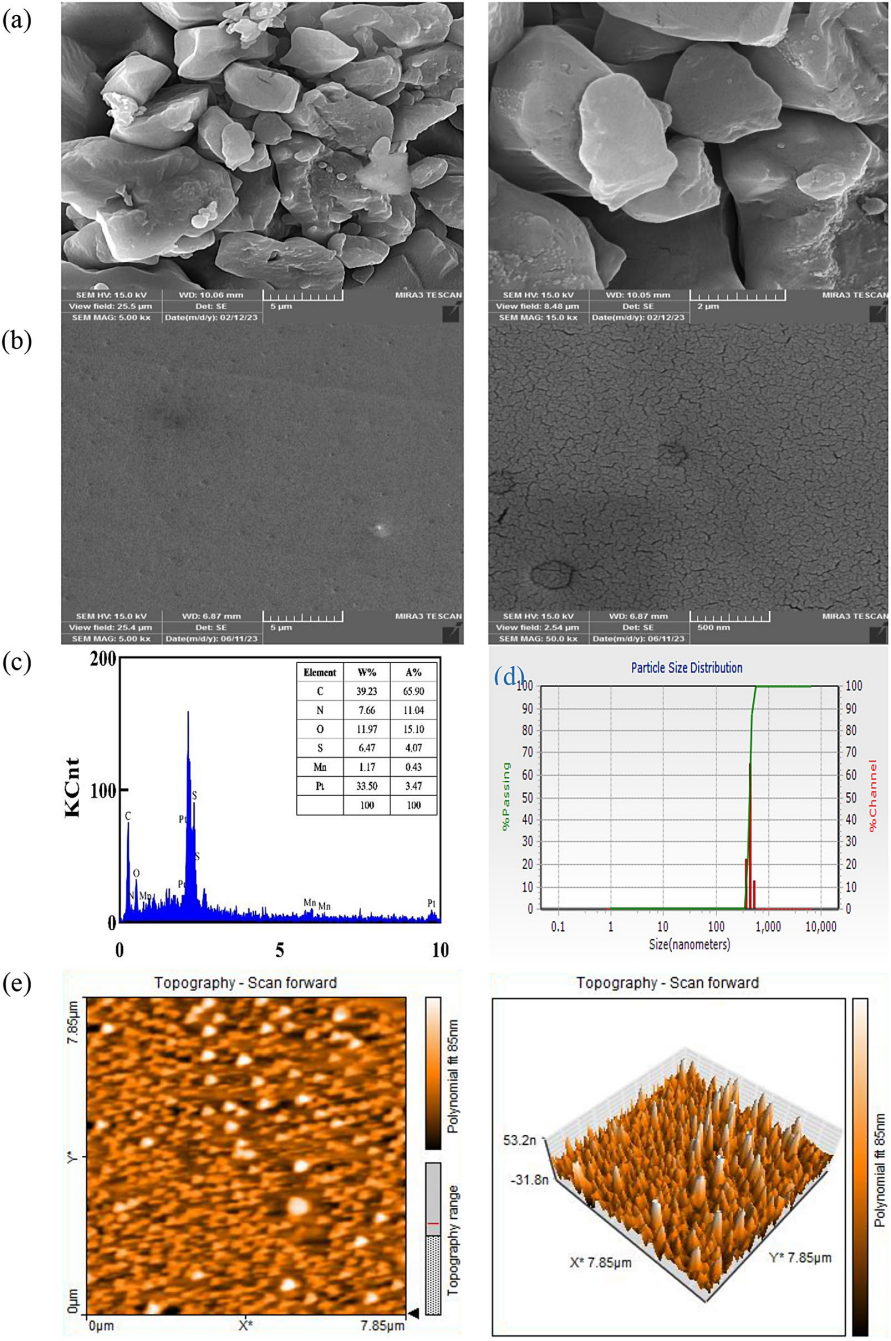
FESEM images of (**a**) Mn(TPDCA)_2_ complex, (**b**) Mn(TPDCA)_2_/CS@Pt NCs-modified GCE electrode, (**c**) EDX analysis of Mn(TPDCA)_2_/CS@Pt NCs-modified GCE electrode, (**d**) DLS, and (**e**) AFM analysis of Mn(TPDCA)_2_

The morphology of the Mn(TPDCA)_2_ complex and the Mn(TPDCA)_2_/CS@Pt NCs-modified GCE were investigated using FESEM (Fig. 1a and b). As seen, various forms of the morphology of Mn(TPDCA)_2_ are exhibited as a result of the complex formation between Mn and TPDCA. Following the electropolymerization process of CS@Pt NCs nanocomposite and casting of the Mn(TPDCA)_2_ on the surface of the GCE, a homogenous layer was successfully established. This layer effectively works as a protective barrier against the accumulation of unwanted substances (physical antifouling) and allows the reaching of AMD molecules toward the electrode surface for subsequent electrochemical reactions [25, 39] The uniformity and stability of this layer play an essential role in enhancing the efficiency and reliability of the electrochemical reactions. Furthermore, the EDX analysis of the modified GCE indicates that it comprises approximately 39.2% C, 33.5% Pt, and 1.17% Mn, approving the uniform construction of Mn(TPDCA)_2_/CS@Pt NCs-modified GCE (Fig. 1c). Figure 1d exhibits the distribution of particle sizes. The average size of the synthesized composite was measured to be 428 nm, with a polydispersity index of 0.0566. The determination of the zeta potential yielded a value of − 97.1 mV. The Mn(TPDCA)_2_ functional groups were also investigated using FT-IR spectroscopy. The following FT-IR bands were obtained: 3434 cm^− 1^ (OH stretching), 3038 cm^− 1^ (aromatic C-H stretching), 1733 cm^− 1^ (C = O from carboxylic acid), 1658 cm^− 1^ (amide group stretching), 1523 cm^− 1^ (C-N stretching), 1434 cm^− 1^ (C-O stretching), 1212 cm^− 1^ (C-O stretching of acyl group), 1076 cm^− 1^ (C-N stretching), and 680 cm^− 1^ (S-C bonding) [32]. Minor changes in asymmetric stretch vibration suggested the Mn coordination with the carboxylate group, indicativing a weak electrostatic interaction between carboxylate and Mn. The topography of the Mn(TPDCA)_2_ complex, which exhibits a rod-shaped morphology, is determined through an AFM technique. The image provides a characteristic depiction of the complex, emphasizing its structural features and overall shape. The AFM technique offers a high-resolution and detailed view of the complex, enabling a better understanding of its physical properties and potential applications (Fig. 1e).

### Surface modification of GCE with CS and Pt NCs

**Fig. 2:**
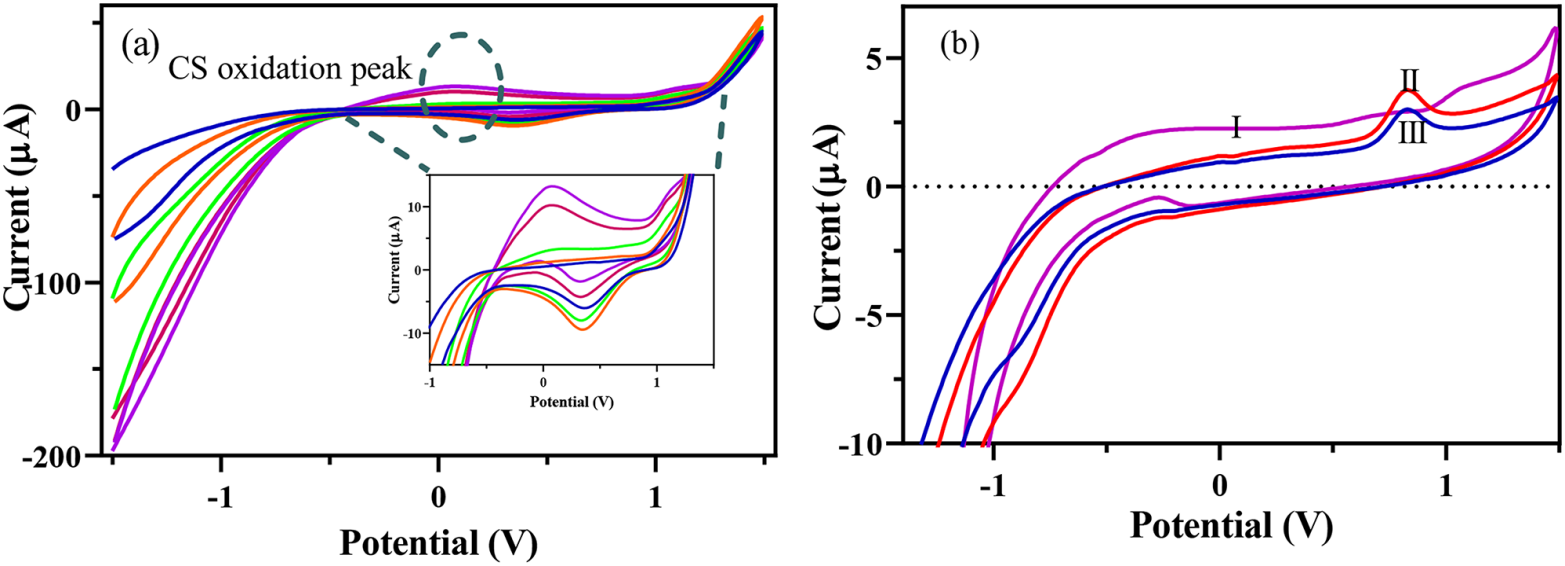
(**a**) CVs of electropolymerization of CS@Pt NCs on GCE (*n* = 5 cycles, scan rate = 100 mV/s) and (**b**) CVs of Mn(TPDCA)_2_/Pt NCs/poly-CS/GCE modified electrode phosphate buffer as supporting electrolyte (I), Mn(TPDCA)_2_/Pt NCs/poly-CS/GCE (II) in the 25 mg/L AMD, and 50 mg/L AMD (III) in the phosphate buffer as supporting electrolyte (50 mM, pH 7.5)

An electropolymerization approach was employed for the surface modification of the GCE. The electropolymerization of CS is a widely used approach for the modification of the surface of electrodes to improve their electrochemical behavior [40–42]. CS moleculs provide a biocompatible thin layer that cantains functional amine groups, making it an special substrate for the next functionalization (Fig. 2a). Furthemore, CS combined nanomaterials were employed as a stabilizing molecule in sensor fabrication because of some outstanding features including easy 3D network-forming capability, high mechanical strength, easy surface modifications, high water permeability, etc. As a mechanism of electropolymerization of CS onto the GCE, amine and hydroxle groups of CS molecules undergo electrochemical oxidation reactions at the given potential range, leading to the production of various free radicals. These free radicals produce polymer chain onto the GCE surface via radical coupling reaction, forming a CS thin layer on the surface of GCE [40]. By mixing CS and Pt NCs, a homogeneous mixture is formed not only through electrostatic bonds but also via dative bond between amine and alcohol groups, trapping inside the 3D structure of polychitosan. The amine (-NH_2_) and alcohol (-OH) groups of CS can carry an nonbonding ione pairs, bonding with Pt NCs via dative interation. Also, PVP of PVP-capped Pt NCs can interact with the hydroxyl group of CS to provide a weak hydrogen bond via molecular rearrangement. When Mn(TPDCA)_2_ is added to the Pt NCs-CS/GCE, this complex can interact with the Pt atoms in the CS layer and connects to the electrode surface through the acidic functional groups on TPDCA. The electropolymerization of CS continues until the preferred thickness of CS film is accomplished. The stability of the CS film and growth rate can be affected by various parameters such as pH, applied potential range, number of CVs, CS concentration, etc. As shown in Fig. 2a, CV peak currents have increasing (in oxidation) and decreasing (in reduction) trends with increasing CV scans, forming an electroconductive thin layer of CS@Pt NCs on the GCE surface. Despite beneficial features, electropolymerization of CS on the surface GCE can result in a decrease in the electrical conductivity of the modified surface. To resolve this problem, different metallic or polymeric materials like Pt NCs are co-electropolymerized on the GCE surface (Fig. 2b). The co-electropolymerization of CS and Pt NCs not only improves the electrical conductivity but also can offer the advantages of enhanced stability and increased specific surface area (Fig. 2b (I)). After the modification of the surface of CS@Pt NCs-modified GCE with Mn(TPDCA)_2_, an oxidation peak appeared around 0.86 V, which corresponds to the oxidation of AMD. This may result in a dual signal amplification strategy involving Mn and Pt metallic counterparts. Also, this peak is another proof of the successful surface modification of CS@Pt NCs-modified GCE with Mn(TPDCA)_2_ (Fig. 2b (II)). Although the current at 0.86 V is decreased in the presence of AMD, the sensitivity is not sufficient for detecting AMD at low levels (Fig. 2b (III)).

**Scheme 1:**
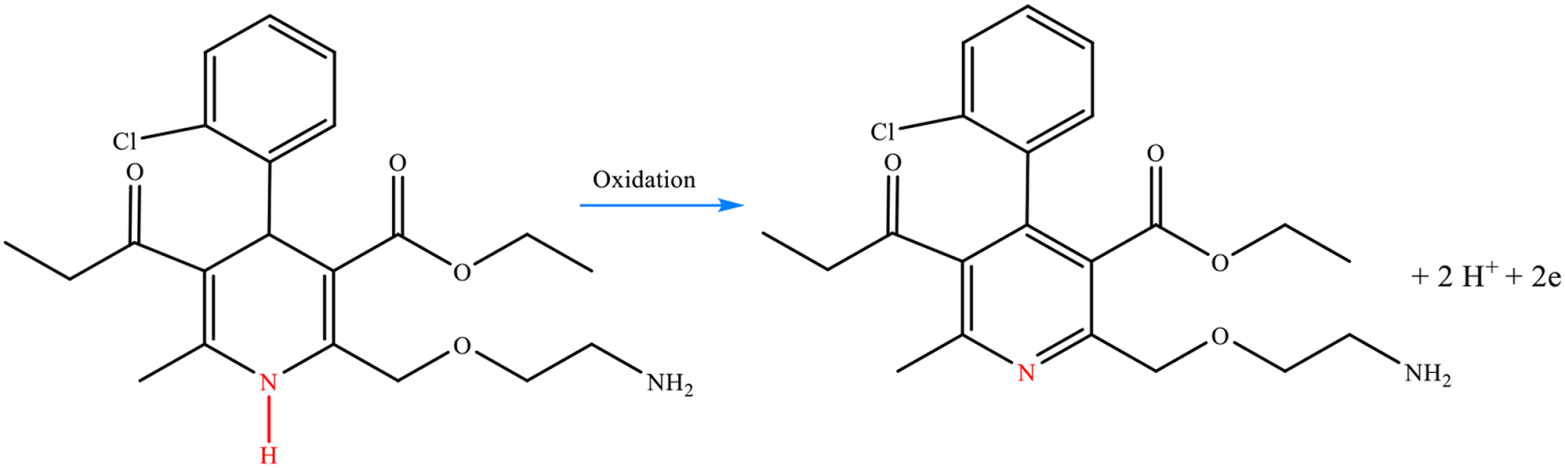
Electrochemical oxidation mechanism of AMD

The amine group of AMD is oxidized to produce a pyridine, involving two electrons and two protons (Scheme 1). This electrochemical oxidation (ECO) is an irreversible reaction that occurs on the surface of the Mn(TPDCA)_2_/CS@Pt NCs-modified GCE. The oxidation of AMD results in the producing a pyridine structure, which has better stability than the amine groups.

### Effect of pH

**Fig. 3:**
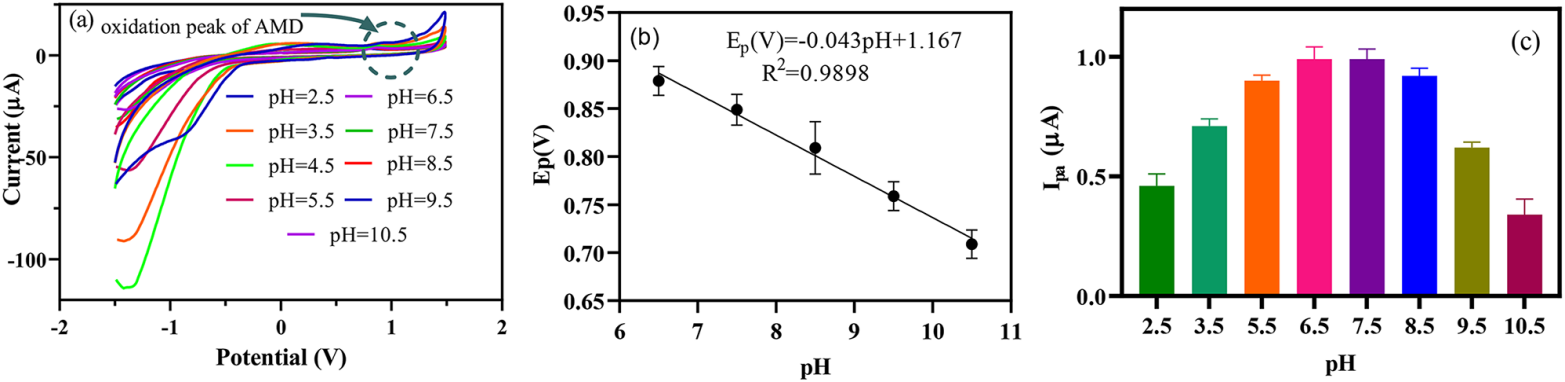
(**a**) CVs of Mn(TPDCA)_2_/CS@Pt NCs-modified GCE at various pHs from 2.5 to 10.5, (**b**) E_p.a._vs. pH curve for the electro-oxidation of AMD, and (**c**) effect of pH on the current intesity. (PBS, 50 mM, potential range – 1.5 to 1.5 V, scan rate 100 mV/s, AMD, 1 mg/L)

Regarding the presence of the ester and amine groups of AMD, the pH can affect its electrochemical activity. Therefore, the effect of pH on the peak current and potential was studied using CV and DPV techniques. Figure 3a displays the CVs in the 2.5 to 10.5 pH range, showing a shifting of AMD oxidation peak position to the negative potentials. The peak potential (E_p.a._) *vis* pH is presented by the following equation (Fig. 3b).

$$\:{E}_{pa}\left(V\right)\:=-0.043\:pH\hspace{0.17em}+\hspace{0.17em}1.167,\:{R}^{2}\hspace{0.17em}=\hspace{0.17em}0.989$$

The slope of the equation is 0.043 V/pH which is close to the 0.0592 V/pH theoretical value, suggesting that the number of electron and proton transformations is equal in the electro-oxidation of AMD. The AMD oxidation peak current (Ipa) of Mn(TPDCA)_2_/CS@Pt NCs-modified GCE was its maximum level at pH = 7.5, proposing the optimum pH for the determination of AMD (Fig. 3c).

### Kinetic study of the oxidation of AMD

**Fig. 4:**
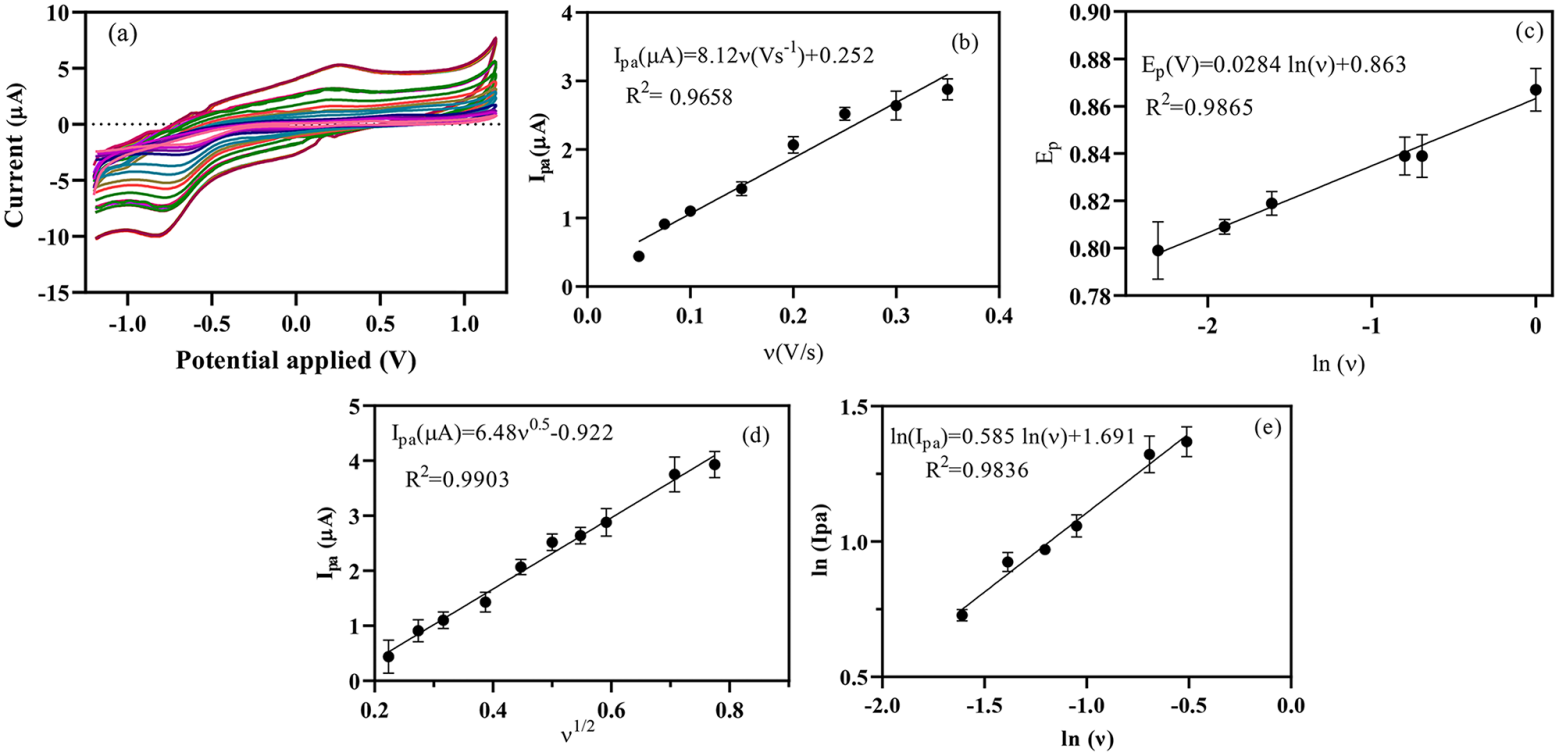
(**a**) CVs at various scan rates, (**b**) I_p.a._ vis. ν, (**c**) E_p_ vis. ln (ν), (**d**) I_p.a._ vis. ν^0.5^, and (**e**) ln (I_p.a._) vis. ln (ν) of Mn(TPDCA)_2_/CS@Pt NCs-modified GCE (PBS, 50 mM, potential range, -1.2 to 1.2 V, AMD, 1 mg/L)

The mechanism of electrochemical activity of AMD on the surface of Mn(TPDCA)_2_/CS@Pt NCs-modified GCE was determined by recording consecutive CVs at various scan rates (Fig. 4a). Upon increasing the scan rate, the peak currents increase correspondingly because of the decreasing of diffusion layer size at the higher scan rates. Consecutive scan rates of the Mn(TPDCA)_2_/CS@Pt NCs-modified GCE were measured in the presence of AMD with a concentration of 20 µg/mL. The oxidation peak of AMD was detected between + 0.75 and + 0.9 V at scan rates of 50–1000 mV/s. The correlation of peak current *vis* scan rates suggests a linear relationship at low scan rates (Fig. 4b), proposing a surface-determined electron transfer (adsorption-controlled mechanism) between the Mn(TPDCA)_2_/CS@Pt NCs-modified GCE and supporting electrolyte at the low scan rates. The I_p.a._*vis* ν for AMD electrooxidation is represented as I_p.a._ (µA) = 8.119ν + 0.2518 (R^2^ = 0.9658) with proper correlation, confirming surface-determined electron transfer [43, 44].

The nature of the reaction, i.e. reversible or irreversible behavior of the AMD oxidation, was checked using E_p.a._*vis* ln (ν) at the scan rates ranging from 50 to 950 mV/s (pH 7.5, 50 mM). In the Mn(TPDCA)_2_/CS@Pt NCs-modified GCE, the peak potential was correspondingly altered with increasing ln (ν), confirming the irreversible and diffusion-controlled electrochemical oxidation of AMD. Also, the peak potential has shifted to a higher positive value. E_p.a._*vis* ln (ν) equation could be presented as E_p.a._(V) = 0.0283 ln (ν) (Vs^− 1^) + 0.8632 (R^2^ = 0.9865).

The Laviron theory, Laviron equation, or Laviron’s model, provides a mathematical method to describe the mechanism of action and kinetics of electrochemical activity, particularly the reaction with electron transfer occuring at the interface between electrode and electrolyte. Here is the Laviron equation:

$$\:{E}_{Pa\:}={E}_{0\:}+\frac{RT}{\alpha\:nF}ln\left(\frac{RTk^\circ\:}{\alpha\:nF}\right)+\frac{RT}{\alpha\:nF}lnv$$

E_0_, T, and n are formal potential, temperature, and the number of transferred electrons during an electrochemical reaction, respectively. R is the gas constant (*R* = 8.314 J mol^− 1^ K^− 1^), and F denotes to Faraday constant (F = 96,493 C mol^− 1^). Also, α and k° constants are the electron transfer coefficient, and standard rate constant of the reaction, respectively. The αn was calculated using the slope E_p.a._*vis* ln (ν) equation with a calculated value of 0.91. Thus, approximately two electrons (1.82 ~ 2) were used in the electro-oxidation of AMD on the surface of Mn(TPDCA)_2_/CS@Pt NCs-modified GCE.

The ln (I_p.a._) *vis* ln (ν) and I_p.a._*vis* ν^0.5^ were plotted to further investigate the mechanism of electro-oxidation of AMD on the Mn(TPDCA)_2_/CS@Pt NCs-modified GCE surface (Fig. 4b and d). The intercept of the I_p.a._*vis* ν^0.5^ plot is near 1.0, proposing diffusion-controlled oxidation of AMD on the Mn(TPDCA)_2_/CS@Pt NCs-modified GCE at the high scan rates. However, the slope ln(I_p.a._) *vis* ln (ν) equation, i.e., ln(I_p.a._) = 0.5851 ln (ν) + 1.691 with R^2^ of 0.9836 (Fig. 4e), is near to 0.5 that doubly confirmed the diffusion-controlled oxidation of AMD on the Mn(TPDCA)_2_/CS@Pt NCs-modified GCE. Also, the surface coverage (Γ*) was calculated by plotting I_p.a._*vis* ν which is expressed by Faraday’s law.

$$\:{l}_{p}=\frac{nFQv}{4RT}=\frac{{n}^{2}{F}^{2}A{{\Gamma\:}}^{*}{\upnu\:}}{4RT}$$

Here, A and Γ^*^ denote surface area and AMD surface amounts, respectively. The calculated values for n and A are 2 and 3.14 × 10^− 2^ cm^2^, respectively. These values could be utilized for the calculation of Γ* with a calculated value of 6.88 × 10^− 5^ mol/cm^2^ for the oxidation of AMD. These results demonstrate the careful diffusion of AMD on the surface of Mn(TPDCA)_2_/CS@Pt NCs-modified GCE.

### Analytical performance

**Fig. 5:**
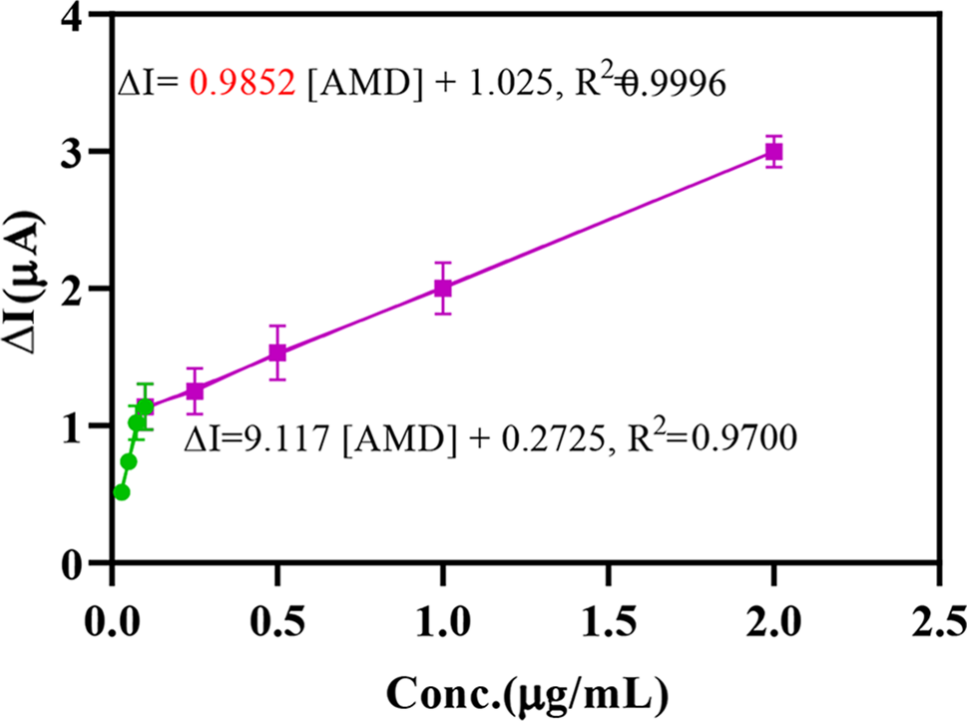
Calibration curve of the Mn(TPDCA)_2_/CS@Pt NCs-modified GCE probe for the detection of AMD in unprocessed plasma media ([Fe(CN)_6_]^−3/−4^, 5 mM)

Under optimal conditions, the calibration curve was plotted using [Fe(CN)_6_]^−3/−4^ as a supporting electrolyte. The key factors for evaluating the analytical performance of Mn(TPDCA)_2_/CS@Pt NCs-modified electrode are the limit of detection (LOD) and limit of quantification (LOQ), which are calculated using the calibration curve. The DPV method was utilized to record the signal of various concentrations of AMD. An increase in the concentration of AMD resulted in a decrease in the peak current of [Fe(CN)_6_]^−3/−4^. Upon the addition of the AMD and reaching the surface of the Mn(TPDCA)_2_/CS@Pt NCs-modified GCE electrode, the electron transfer is hindered by the AMD molecules, resulting in a decrease in the oxidation current of [Fe(CN)_6_]^−3/−4^. The linear range and lower limit of detection of the probe were 53 nM-3.5 µM and 53 nM, respectively. The developed probe response toward the AMD concentrations was linear in two ranges with equations of ΔI(µA) = 9.117 [AMD] + 0.2725 (R^2^ = 0.970) and ΔI(µA) = 0.985[AMD] + 1.025 (R^2^ = 0.9996) (Fig. 5). In this study, ΔI was considered as the analytical signal, which is defined as the difference between current of probe in the absence and presence of AMD. The results confirmed that the Mn(TPDCA)_2_/CS@Pt NCs sensor has a lower LOD and a broader linear range compared to reported sensors. Table 1 summarizes the analytical features of previously developed methods for the determination of AMD.

**Table 1:** Analytical performances and media of the reported approaches for the quantification of AMD

Materials	Technique	LOD	LOQ	Media	Ref
CNTs/Ag/NH_2_-CNTs	SWASV	77.6 fM	258.6 fM	biological and pharmaceutical samples	[29]
GL Pt-doped NiCo_2_O_4_/SPE	DPV	0.009 µM	0.027 µM	urine, serum and drug	[5]
Aniline-co-anthranilic acid	CV	2.66 pM	8.78 pM	plasma and serum	[17]
MnC_4_Pc-ZnO/ITO	CV	20 nM	-	-	[30]
NiMoO_4_/CS	DPV	4.62 nM	24.65 nM	Pharmaceutical Formulation and Human Serum	[27]
AuNRs-GO-CNTs	DPV	3.0 nM	10.0 nM	human blood serum and plasma	[34]
Mn(TPDCA)_2_/CS@Pt NCs	DPV	53 nM-3.5 µM	53 nM	plasma	This work

^*Square Wave Anodic Stripping Voltammetry (SWASV), Indium tin oxide (ITO)^

### Method validation

#### Accuracy, inter-day, and intra-day repeatability

To evaluate the intra-day and inter-day precisions, three levels of AMD concentrations from the calibration range were selected. Table 2 presents the repeatability data for both inter-day and intra-day assessments. Furthermore, the accuracy of the developed method was determined in plasma media. The collected data points exhibit acceptable levels precision and accuracy of the method. The repeatability measurements, expressed as relative standard deviation (RSD%), varied from 16.0 to 8.3%, while the accuracy percentages, expressed as relative error (RE%), ranged from − 1.6% to -4.5% with recoveries ranging from 94.5 to 104.7%. The findings indicate that this method can analyze AMD in plasma samples and yield reliable results.

**Table 2:** Accuracy, interday- and intraday precision of the developed electrochemical method for the detection of AMD in plasma samples

Nominal concentration (µg/mL)	Intraday precision (RSD%)	Interday precision (RSD%)	Interday accuracy (RE%)	Recovery (%)
0.05	13.0	16.0	-1.6	98.4
1.0	9.2	13.1	+ 4.7	104.7
2.5	8.3	10.1	-4.5	94.5

#### Specific of Mn(TPDCA)2/CS@Pt NCs-modified GCE probe toward AMD recognition

**Fig. 6:**
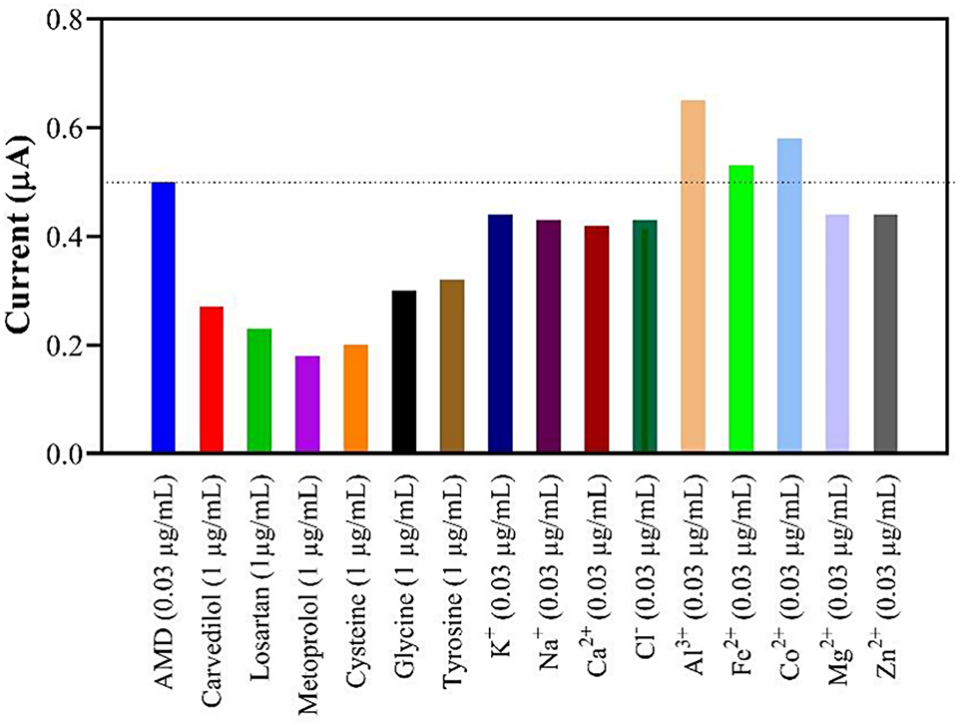
Effect of some common interfering agents on the detection of AMD in plasma samples

The ability of the modified electrode to selectively determine the AMD concentration in the presence of various interfering agents was evaluated using the DPV method. The anti-interference capability of the constructed electrochemical sensor has been investigated using ions (K^+^, Na^+^, Ca^+^, Cl^−^, Al^3+^, Fe^2+^, Co^2+^, Mg^2+^, Zn^2+^), amino acids (glycine, cysteine, tyrosine), and medicines (carvedilol, metoprolol, losartan). The experimental results indicate the sensor has a high selectivity under typical conditions, and the probe signal is unaffected by the interfering substances. It detects low concentrations of AMD in the presence of these agents even at concentrations higher than the biological levels (Fig. 6).

### Application of the probe

#### Application in spiked samples

The fabricated Mn(TPDCA)_2_/CS@Pt NCs-modified GCE platform was employed for the quantification of AMD in unprocessed plasma samples. Under optimal conditions, three different concentrations of AMD (0.25, 0.1, and 0.05 µg/mL) were spiked to the drug-free plasma samples and determined using the calibration curve. The recovery was calculated as a parameter for assessing the accuracy of the detection ability of the probe. The calculated recoveries were 98.4%, 100.3%, and 97.4% for 0.5, 0.10 and 0.25 µg/mL, respectively. These results show the applicability of the developed probe for the detection of AMD in clinical samples without any need for sample pretreatment steps.

#### Determination in real samples

The real potential of the probe for determining AMD must be thoroughly examined in the patient’s samples which is well-known as real samples. These samples were used to detect the AMD to evaluate its practicality. For any experiment involving human volunteers, informed consent forms were filled out by the donners (license code: IR.TBZMED.REC.1402.724). The Mn(TPDCA)_2_/CS@Pt NCs/GCE was used to determine the presence of AMD in the samples. The calibration curve was used to calculate the concentration of AMD in real samples after applying optimal conditions. The concentrations of AMD found in the patient’s plasma samples (#9 pateint) is collected in Table 3. The obtained results showed that the concentration range is from 0.207 to 1.479 µg/mL.

**Table 3:** Determination of AMD in patient’s plasma samples using the developed probe

Patient (#)	Gender	Measured concentration (µg/mL)
1	Female	0.233
2	Female	0.261
3	Female	N.D
4	Male	0.207
5	Male	N.D
6	Female	1.479
7	Male	0.789
8	Female	N.D
9	Female	0.687

## Conclusions

An innovative probe was fabricated for the detection of AMD in plasma samples using Mn(TPDCA)_2_/CS@Pt NCs-modified GCE probe. Pt NCs provide a high surface area and enhance the sensitivity of the probe by increasing the electrical conductivity of the probe. The effect of pH on AMD detection is explored, providing valuable insights into the behavior of the sensor under different environmental conditions. CS@Pt NCs were deposited on the surface of the electrode to increase the electrode stability and diminish the reaching of the interfering substances onto the surface of the probe. In addition, Mn(TPDCA)_2_ was used as a signal amplifying agent. The dynamic range and lower limit of quantification (LLOQ) of the developed method were 53 nM-3.5 µM and 53 nM, respectively, covering a broad range of potential AMD concentrations in plasma. This probe demonstrated strong intra-day and inter-day repeatability with RSD% values ranging from 8.3 to 16.0%, along with favorable selectivity for AMD in the presence of common interfering chemicals. In addition to the excellent analytical merits of the Mn(TPDCA)_2_/CS@Pt NCs-modified GCE, this probe proposes a low-sample pretreatment approach for the detection of AMD in patient plasma samples. However, the main limitation of the probe is the complex synthesis process of Mn(TPDCA)_2_ and the challenge of preparing stable suspension with CS molecules. Regarding all the obtained optimization and validation results, this probe has the potential to be a rlible probe for AMD concentration determination in real samples.

## Data Availability

No datasets were generated or analysed during the current study.

## Abbreviations

AMD: Amlodipine
HPLC: High-performance liquid chromatography
GC: Gas chromatography
CE: Capillary zone electrophoresis
TDM: Therapeutic drug monitoring
DI: Deionized
ECO: Electrochemical oxidation
EDX: Dispersive X-ray analysis
PVP: Polyvinylpyrrolidone
EG: Ethylene glycol
Pt NCs: Platinum nanocubes
CS: Chitosan
TPDCA: 5-oxo-2,3-dihydro-5 H-[1,3]-thiazolo [3,2-a] pyridine-3,7-dicarboxylic acid
GCE: Glassy carbon electrode
DPV: Differential pulse voltammetry
LOD: Limit of detection
LOQ: Limit of quantitation
LLOQ: Lower limit of quantification
RSD: Relative standard deviation
RE: Relative error
